# Targeting CCN Proteins in Rheumatoid Arthritis and Osteoarthritis

**DOI:** 10.3390/ijms22094340

**Published:** 2021-04-21

**Authors:** Iona J. MacDonald, Chien-Chung Huang, Shan-Chi Liu, Yen-You Lin, Chih-Hsin Tang

**Affiliations:** 1 Graduate Institute of Basic Medical Science, Collage of Medicine, China Medical University, Taichung 40402, Taiwan; ionamac@gmail.com (I.J.M.); chas6119@gmail.com (Y.-Y.L.); 2 School of Medicine, Collage of Medicine, China Medical University, Taichung 406040, Taiwan; u104054003@cmu.edu.tw; 3 Division of Immunology and Rheumatology, Department of Internal Medicine, China Medical University Hospital, Taichung 404332, Taiwan; 4 Department of Medical Education and Research, China Medical University Beigang Hospital, Yunlin 65152, Taiwan; sdsaw.tw@yahoo.com.tw; 5 Graduate Institute of Biomedical Sciences, Collage of Medicine, China Medical University, Taichung 406040, Taiwan; 6 Chinese Medicine Research Center, China Medical University, Taichung 406040, Taiwan; 7 Department of Biotechnology, College of Health Science, Asia University, Taichung 413305, Taiwan

**Keywords:** CCN proteins, CCN family, rheumatoid arthritis, osteoarthritis, juvenile idiopathic arthritis

## Abstract

The CCN family of matricellular proteins (CYR61/CCN1, CTGF/CCN2, NOV/CCN3 and WISP1-2-3/CCN4-5-6) are essential players in the key pathophysiological processes of angiogenesis, wound healing and inflammation. These proteins are well recognized for their important roles in many cellular processes, including cell proliferation, adhesion, migration and differentiation, as well as the regulation of extracellular matrix differentiation. Substantial evidence implicates four of the proteins (CCN1, CCN2, CCN3 and CCN4) in the inflammatory pathologies of rheumatoid arthritis (RA) and osteoarthritis (OA). A smaller evidence base supports the involvement of CCN5 and CCN6 in the development of these diseases. This review focuses on evidence providing insights into the involvement of the CCN family in RA and OA, as well as the potential of the CCN proteins as therapeutic targets in these diseases.

## 1. Introduction

Of the more than 100 different types of arthritis, rheumatoid arthritis (RA) and osteoarthritis (OA) are two of the most common [1]. The chronic inflammation and autoimmunity associated with RA disease principally targets the synovium, provoking the membrane lining to produce synovial fluid that causes synovitis and joint pain, and ultimately chronic and progressive joint erosion [2]. OA is a whole-joint disease involving the increased remodeling of the articular cartilage, subchondral bone and bone marrow compartments, as well as the synovium, during its onset and progression [3]. Although these are two distinct arthritis diseases, some similar clinical and pathological manifestations exist, such as joint stiffness, synovial inflammation, destruction of the articular cartilage and bone erosion [4]. Treatment remains a significant clinical challenge in these diseases. In spite of the recent emergence of targeted therapies and immune-modulating agents for RA, a sizeable proportion remain treatment-refractory and experience increasing clinical impairment and even premature mortality [5,6,7]. Moreover, no disease-modifying treatments exist as yet for OA [8]. Enhancing our understanding about the ways in which CCN proteins affect the pathophysiological processes of these forms of arthritis may lead to future treatment strategies that target the functions and mechanisms of action of these proteins, and effectively alleviate patients’ suffering.

The CCN family consists of six matricellular proteins, cysteine-rich 61 (CYR61/CCN1), connective tissue growth factor (CTGF/CCN2), nephroblastoma-overexpressed (NOV/CCN3), Wnt-1 induced secreted protein-1 (WISP1/CCN4), Wnt-1 induced secreted protein-2 (WISP2/CCN5) and Wnt-1 induced secreted protein-3 (WISP3/CCN6), all of which are essential players in the key pathophysiological processes of angiogenesis, wound healing and inflammation [9]. They are well recognized for their important roles in many cellular processes including cell proliferation, adhesion, migration and differentiation, and the regulation of extracellular matrix (ECM) differentiation [9]. Much evidence has implicated four of the proteins (CCN1, CCN2, CCN3 and CCN4) in the inflammatory pathologies of RA [10,11,12,13] and OA [14,15,16,17,18,19,20]; the evidence base is smaller for CCN5 and CCN6 in these diseases [21,22,23,24]. Interestingly, although much similarity exists among the primary structures of the CCN proteins, the considerable differences identified in their three-dimensional structures result in distinctly different protein interactions and known binding partners that result in distinctly different functions [25]. Moreover, interactions between CCNs enable the regulation of cellular function and various receptors including insulin-like growth factors, heparan sulfate proteoglycans and integrins, among others [25]. Finally, the CCN proteins are vital contributors to the biological processes mentioned above (angiogenesis, adhesion, migration and differentiation, ECM remodeling, cartilage growth and maintenance, wound healing and inflammation) [25].

**Figure 1:**
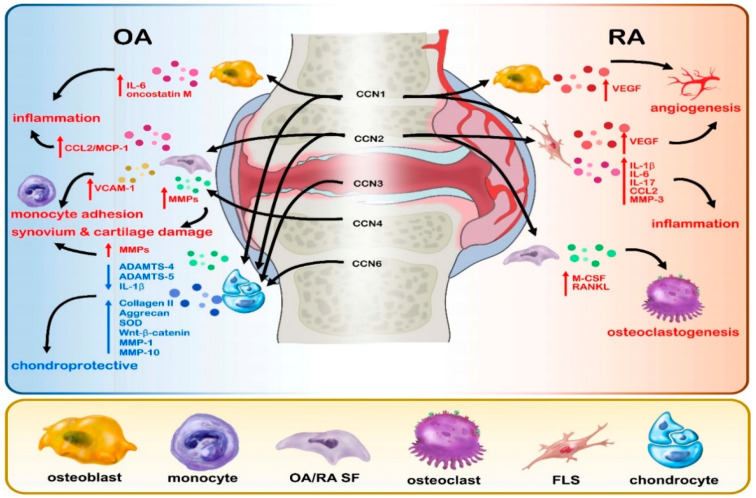
Graphical representation of the effects of CCN proteins 1–6 in RA and OA disease.

This review discusses the evidence regarding the involvement of CCN proteins in RA and OA (see Table 1 and Figure 1).

## 2. CCN1 in RA and OA

CCN1 can play an important and harmful role in OA disease by promoting the production of inflammatory cytokines such as interleukin 6 (IL-6) and oncostatin M in human osteoblasts through integrin-dependent signaling [26]. Furthermore, CCN1 overexpression accelerates inflammation and matrix degradation in human OA cartilage [28]. *Ccn1* messenger RNA (mRNA) and *ADAMTS-4* (a disintegrin and metalloproteinase with thrombospondin motif 4) mRNA is significantly upregulated in human OA cartilage tissue compared with normal, non-OA cartilage [15,27]. Moreover, *Ccn1* and *ADAMTS-4* mRNA expression is positively correlated in OA cartilage tissue, while levels of CCN1 and ADAMTS-4 protein expression are markedly upregulated in OA chondrocytes, compared with those in normal chondrocytes [27]. CCN1 stimulates the proliferation of OA chondrocytes through ADAMTS-4 [27]. Intriguingly, CCN1 activities can also be beneficial in OA. The binding of CCN1 with ADAMTS-4 enables CCN1 to suppress ADAMTS-4 aggrecanase activities, which are critical to the development of OA disease [15,27]. Notably, IL-1β promotes ADAMTS-4 and inhibits CCN1 expression [27]. CCN1 expression in OA chondrocytes is also inhibited by IL-1α [15] and upregulated by transforming growth factor beta (TGF-β) [27]. Interestingly, TGF-β-treated human OA chondrocytes have demonstrated aggrecanase activity after knockdown of CCN1 expression, which suggests that modulating CCN1 and TGF-β activity may promote the repair of OA cartilage [27]. Thus, the evidence appears to be conflicting as to the effects of CCN in OA. Further studies are needed to fully understand the relationships.

Chronic synovitis in RA joints results from the persistent production of proinflammatory cytokines IL-1 and tumor necrosis factor alpha (TNF-α) from activated mononuclear cells, inducing cartilage degradation [43]. The chemotaxis, survival and proliferation pathways in mononuclear cells can be activated by chemokines [44], including chemokine ligand 2 (CCL2, also known as monocyte chemoattractant protein-1 (MCP-1), a critical regulator in the process of monocyte migration and infiltration to the site of RA inflammation, as CCL2/MCP-1 is produced at significantly higher levels in blood, synovial fluid and synovial tissue from patients with RA compared with samples from non-RA controls [45,46]. High levels of CCN1 expression are also found in RA synovial fluid compared with synovial fluid from patients without RA [11], while CCN1 expression is minimal in RA hip and knee cartilage and absent in normal hip cartilage [47].

Several lines of evidence demonstrate that CCN1 is a key contributor to the RA disease process. CCN1 induces upregulation of CCL2/MCP-1 expression in osteoblasts, and subsequently, promotes monocyte migration by inhibiting microRNA (miR)-518-5p [11]. As a component of the ECM, CCN1 plays a role in endothelial cell adhesion, migration, proliferation, and differentiation [48]. CCN1 interacts with IL-17 to promote fibroblast-like synoviocyte (FLS) proliferation in RA synovial fluid and inhibits FLS apoptosis, contributing to the hyperplasia of synovial lining cells [29]. Attacks by FLS on RA synovial tissue and cartilage implicates CCN1 as a key contributor to the joint erosion and destruction seen in RA disease [29], which is emphasized by subsequent research revealing that CCN1 promotes IL-17 production in RA by upregulating IL-6 in human RA FLS [30]. CCN1 also increases synthesis of the precursor IL-1β (pro-IL-1β) in human RA FLS [31] and upregulates vascular endothelial growth factor (VEGF) expression in osteoblasts, inducing endothelial progenitor cell (EPC)-angiogenesis in RA disease [32].

Much cellular and preclinical evidence has suggested that modulating CCN1 expression in RA disease has therapeutic potential [49]. Promising experimental findings suggest the feasibility of designing peptide-based vaccination against RA. The murine monoclonal antibody (mAb) 093G9 specifically targets CCN1 and effectively antagonizes its effects on the production of pro-IL-1β and matrix metallopeptidase (MMP)-3 expression by FLS [33], while in mice with collagen-induced arthritis (CIA), mAb 093G9 treatment reduces inflammatory reactions and ameliorates joint disease [30]. Structural and functional investigations have delineated the CCN1 epitope that is recognized by 093G9 and defined its epitope specificity, opening up the possibilities for developing mAb drugs and peptide vaccines targeting CCN1 [33].

In summary, CCN1 is both beneficial and harmful for OA and harmful in RA. CCN1 increases the expression of oncostatin M in human osteoblastic cells [26], synthesizes pro-IL-1β and enhances the expression of MMP-3 in human RA FLS [31], promotes FLS proliferation and participates in RA pathogenesis via the IL-17-dependent pathway [49] and also promotes the expression of CCL2 and monocyte migration by inhibiting miR-518-5p expression in osteoblasts via mitogen-activated protein kinase (MAPK) signaling [11].

## 3. The Role of CCN2 in RA and OA

All layers of normal cartilage express CCN2 protein and mRNA in a small percentage of chondrocytes, whereas OA cartilage is characterized by markedly increased numbers of CCN2-positive chondrocytes that correlate with increasingly severe OA disease [50]. Our laboratory has previously described finding high levels of CCL2/MCP-1 expression in OA synovial fibroblasts (OASFs) compared with normal synovial fibroblasts, and we have observed that treating OASFs with CCN2 increases CCL2/MCP-1 expression [34]. OASFs and supernatants from CCN2-treated OASFs promote the migration of monocyte cells via the αvβ5 integrin, focal adhesion kinase (FAK), mitogen-activated protein (MEK), extracellular signal-regulated kinase (ERK), and nuclear factor-kappa B (NF-κB)/AP-1 signaling transduction pathway [34].

Intriguingly, CCN2 can promote proliferation and differentiation of articular chondrocytes without inducing their hypertrophic calcification [51]. Thus, it has been hypothesized that articular chondrocytes promote CCN2 production in OA cartilage, and thereby, increase the cell number and compensate for the deficiency in the ECM [51]. This is supported by research showing that in rats with monoiodoacetic acid (MIA)-induced OA, a single injection of recombinant CCN2 into the joint cavity effectively repaired articular cartilage and ameliorated OA disease, which suggests that CCN2 may help to regenerate articular cartilage [52]. Likewise, transgenic mice that overexpress *Ccn2* in articular cartilage appear to be protected against OA-like degenerative changes in aged knee joint cartilage, which may mean that CCN2 stabilizes articular cartilage [53]. The idea that CCN2 plays a chondroprotective role is supported by findings showing significantly accelerated degeneration of lumbar intervertebral discs (IVDs) in *Ccn2*-knockout mice [35]. An analysis of CCN2 expression in rat IVD cells found that Wnt-β-catenin signaling regulates the *Ccn2* gene and protein via the MAPK pathway, raising the possibility that it could be worth targeting Wnt-β-catenin signaling in preclinical treatment of IVD degeneration [54]. Interestingly, some research has reported that the deletion of *Ccn2* in mice increases articular cartilage thickness and prevents the development of OA in cut cartilage [55], whereas other researchers have found that *Ccn2* deletion in articular chondrocytes of male transgenic mice fails to protect them from developing post-traumatic osteoarthritis [56]. In brief, most research supports the potential beneficial role of CCN2 in OA.

In regard to RA disease, CCN2 is strongly expressed in the matrix and perivascular cells in RA human hip and knee synovium samples, as well as in chondrocytes from RA hip and knee cartilage [47]. In normal human hip synovium, CCN2 is moderately expressed in the superficial layers, matrix and perivascular cells, and weakly expressed in normal hip cartilage [47]. The wide-ranging biological activity of CCN2 is characterized by inflammatory, wound healing and profibrotic activity [57,58], proangiogenic activity [57], protumorigenic activity [59] and the promotion of endochondral ossification [60]. In relation to RA, serum CCN2 concentration has shown significant discriminative ability and superior diagnostic performance compared with the rheumatoid factor (RF) and anti-citrullinated protein antibodies (ACPA) assays, which lack sensitivity and specificity [61]. Serum CCN2 discriminates RA from other rheumatic diseases, such as ankylosing spondylitis, gout, systemic lupus erythematosus and OA [61]. Serum CCN2 concentrations are higher in patients with active RA compared with CCN2 concentrations in normal healthy controls and patients with inactive RA disease; CCN2 also promotes articular destruction in RA by increasing osteoclastogenesis [36] and FLS proliferation in RA [62]. Investigations suggest that CCN2 acts synergistically with macrophage colony-stimulating factor (M-CSF) and receptor activator of nuclear factor-kappa B ligand (RANKL) to promote osteoclastogenesis and that excessive CCN2 production by RA synovial fibroblasts (RASFs) enhances osteoclastic function through integrin αVβ3-mediated pathways such as FAK and ERK1/2 phosphorylation [36]. CCN2 contains four distinct modules that are connected in tandem-insulin-like growth factor-binding protein (IGFBP)-like, von Willebrand factor (vWF) type C repeat, thrombospondin type 1 (TSP-1) repeat and carboxyl-terminal (CT) modules [63]. Inhibiting each of these modules with mAbs neutralizes the effect of CCN2 in human RA synovial cells [63]. In vivo investigations suggest that targeting CCN2 function may be beneficial in RA, as arthritis was significantly ameliorated in CIA mice administered neutralizing anti-CTGF mAb, which effectively suppressed pathologic proliferation of T lymphocytes and restored aberrant osteoclastogenesis [64].

A proteomics analysis has confirmed the importance of angiogenesis in RA progression, by demonstrating the upregulation of CCN2 and other vasculature development-related proteins in cultures of FLS from patients with RA compared with FLS from healthy normal controls [37]. Furthermore, *Ccn2* and *VEGF* mRNA and protein expression are markedly downregulated in RA FLS transfected with CCN2 knockdown, while recombinant human CCN2 significantly enhances the proliferation and migration of human umbilical vein endothelial cells (HUVECs) in Transwell assays [37]. Interestingly, cellular studies have implicated CCN2 in the regulation of MMP expression in RASFs [65]. After subjecting RASFs to 24 h of 10-hydroxy-2-decenoic acid (10H2DA) treatment, CCN2 expression was downregulated and subsequent investigations found that as CCN2 expression decreased in RASFs, so did levels of MMP expression [65]. Notably, several endogenous anti-inflammatory/proresolution lipid mediators are capable of accelerating the resolution of inflammation [66]. These proresolving mediators include resolvins, which are derived from the polyunsaturated omega-3 fatty acids docosahexanoic acid (DHA) or eicosapentanoic acid (EPA) [66]. The D- and E-series resolvins exhibit potent anti-inflammatory/proresolution effects in animal models of inflammation [66]. In particular, one study has demonstrated that resolvin D1 (RvD1) effectively decreases pannus formation and reduces cartilage damage in CIA mice by suppressing concentrations of CCN2 and proinflammatory cytokines in serum and RA FLS, through the upregulation of miR-146a-5p [67]. The above studies mostly confirm the aggravating character of CCN2 in RA.

In summary, CCN2 is beneficial for OA, but possibly harmful for RA. For instance, CCN2 may regenerate OA articular cartilage [52] and help to stabilize the matrix, since *Ccn2* overexpression in articular cartilage seems to protect against OA-like degenerative changes in transgenic mice [53]. CCN2 may also be chondroprotective, as IVD degeneration is significantly accelerated in *Ccn2*-knockout mice [35]. Research indicating that CCN2 expression in rat IVD cells is regulated by Wnt-β-catenin signaling suggests that targeting Wnt-β-catenin signaling may be worth considering in the treatment of IVD degeneration [54]. In contrast, it appears that the less CCN2 the better in RA, as the downregulation of CCN expression in RASFs is accompanied by decreasing levels of MMP expression [65].

## 4. The Role of CCN3 in RA and OA

Several studies demonstrate the benefits of CCN3 in OA pathogenesis. Investigations into CCN3 functioning in adult mice have revealed that loss of normal CCN3 function impairs the homeostasis of articular cartilage cells in the adult knee joint and leads to severe OA-like pathology in all tissues of the joint, accompanied by high Osteoarthritis Research Society International (OARSI) scores [68]. These investigations are supported by later research showing that CCN3 is present in epiphyseal chondrocytes of newborn rats and in normal articular cartilage of young mice and rats, but is rapidly downregulated in rat knees with MIA-induced OA [69]. The researchers described protective implications of exogenous CCN3 in rats with MIA-induced OA, as CCN3-treated OA knees exhibited less cartilage degeneration according to tidemark integrity scoring and had higher lubricin expression in the articular cartilage compared with untreated knees [69]. Investigations using human and rat OA articular cartilage, as well as an anterior cruciate ligament transection (ACLT) rat model of OA, have demonstrated that recombinant CCN3 or CCN3 overexpression is protective in OA by suppressing IL-1β-induced activation of the PI3K/AKT/mTOR signaling pathway [38]. In another investigation, treating rat IVD nucleus pulposus cells with increasing doses of recombinant CCN3 dose-dependently reduced antiproliferative activity, while TGF-β treatment increased nucleus pulposus cell proliferation, which was not blocked by the addition of CCN3, indicating that TGF-β overrides the antiproliferative function of CCN3 [70]. The trend towards increased TGF-β expression during disc degeneration and reduction in CCN3 expression, accompanied by a simultaneous increase in CCN2 expression, may reflect a reparative response that enhances matrix synthesis and promotes changes in cell numbers [70]. However, in experiments involving cartilage-specific CCN3-overexpressing transgenic mice, researchers have described CCN3-driven degradative changes in aging articular cartilage [71]. In those studies, CCN3-overexpressing articular cartilage was characterized by severe degenerative changes that increased with aging and the increased accumulation of CCN3 appeared to promote chondrocyte senescence [71].

The exact role of CCN3 in RA is uncertain. Expression patterns of CCN3 in human joints confirm the absence of CCN3 in normal hip synovium and cartilage, RA hip or RA knee cartilage and OA hip and OA knee cartilage, while CCN3 is weakly expressed in the superficial layers and matrix of RA knee and OA hip synovium samples [47]. Some researchers have suggested that CCN3 could serve as a disease activity biomarker for RA, with significant positive correlations observed between CCN3 levels and 28-joint Disease Activity Score (DAS28, whether characterized by erythrocyte sedimentation rate [ESR] or C-reactive protein [CRP]), with higher DAS28 scores reflecting worsening disease [13]. Significant positive correlations have also been recorded between CCN3 levels and titers of RA-specific anti-cyclic citrullinated peptide antibody (anti-CCP Ab), and between CCN3 and IL-6 expression; no such associations have been observed between CCN3 and RF, or CCN3 and TNF-α [13].

In summary, CCN3 is beneficial for OA and plays an important role in the development of RA disease. CCN3 levels decrease rapidly after MIA injection in rat OA knees and exogenous CCN3 treatment is associated with less articular cartilage damage in rat OA knees compared with untreated knees [69]. Interestingly, some researchers have reported finding that during degenerative disc disease, TGF-β suppresses CCN3 activity and upregulates CCN2 expression, a phenomenon that may be associated with a reparative response [70]. In RA, higher serum CCN3 correlates with higher DAS28 scores, inflammatory markers and greater disease severity [13].

## 5. The Role of CCN4 in RA and OA

WISP1 may be a useful molecular target in OA. A genetic variation at the *WISP1* gene locus appears to influence spinal OA, with one study reporting that postmenopausal Japanese women with the AA genotype (without the G allele) at the *WISP1* 2364A/G single nucleotide polymorphism (SNP) had significantly higher spinal endplate sclerosis scores compared with women carrying the G allele [72]. The study researchers suggested that performing *WISP1* genotyping could be beneficial in the prevention and management of spinal OA. This notion is supported by a later analysis of differential gene expression profiles in OA cartilage that identified several novel genes implicated in OA pathophysiology [73]. When the researchers combined a mill-based RNA isolation technique with high-density oligonucleotide array analysis to examine differential gene expression patterns of chondrocytes in damaged and intact human cartilage within the same knee OA joints, six genes (including *WISP1*) were found to be upregulated in the lesional cartilage area (not in nonlesional areas) of all patients [73]. None of the six genes had previously been identified as playing a role in the damaging effects of OA joint destruction [73].

Investigations into intracellular signaling pathways have helped to clarify important ways in which CCN4 contributes to OA pathophysiology. In one study, CCN4 stimulation of human OASFs upregulated vascular cell adhesion molecule-1 (VCAM-1) expression via the Syk, PKCδ, JNK, c-Jun and AP-1 signaling pathways, which promoted monocyte adhesion to the OASFs [39]. In another study, expression profiling of Wnt signaling molecules confirmed marked increases in CCN4 expression in human and murine OA cartilage and synovium, and the researchers found that recombinant WISP1 stimulation of macrophages and chondrocytes upregulated MMPs and aggrecanase, apparently independently of IL-1 [40]. They also reported that inoculating the articular joints of naïve mice with WISP1 adenovirus enhanced MMP expression in the synovium and cartilage extracellular matrix damage, independently of IL-1α and IL-1β [40]. In contrast, other researchers suggest that stimulating OASFs with CCN4 induces time- and concentration-dependent increases in IL-6 production via the αvβ5 integrin, PI3K, Akt and NF-κB signaling pathways, emphasizing an important role for IL-6 during OA pathogenesis [74].

A series of investigations by a research group from the Netherlands has examined the implications of Wnt signaling and WISP1 expression in OA pathology [17,18,19,20]. Intra-articular injection of Wnt8a and Wnt16 into murine knee joints increased protease activity in the joint and induced cartilage damage, which was significantly decreased after inhibiting the canonical Wnt signaling pathway with the selective inhibitor Dickkopf-1 (DKK-1) [17]. Moreover, the study evidence linked overexpression of WISP1, a downstream protein of canonical Wnt signaling, to OA-like damage in the cartilage that was similar to that of Wnt8a and Wnt16 overexpression [17]. Interestingly, canonical Wnt signaling did not appear to involve IL-1 [17]. In their 2016 review of evidence implicating the Wnt signaling pathway in OA disease, van den Bosch and colleagues concluded that the complexity of this pathway and its multilayered crosstalk with TGF-β signaling (an important contributor to joint homeostasis) makes it difficult to determine the risk of undesired side effects [18]. Targeting WISP1 for OA therapy seems more feasible, which is supported by study findings showing that it is possible to regulate various aspects of OA pathology without interfering with normal processes in mice lacking *Wisp1*, an experimental model of OA [19]. Subsequent in vitro research by the same study group has confirmed that increased expression of *WISP1* is detrimental for cartilage integrity [20].

Whereas CCN4 is not expressed in normal hip synovium samples and is undetectable in normal hip, RA knee and RA hip cartilage obtained from patients undergoing joint replacement, weak-to-moderate CCN4 expression has been found in the superficial layers, matrix and perivascular cells of OA hip, RA knee and OA knee synovium samples from patients with advanced RA or OA disease [47]. Associations between several *WISP1* SNPs and RA susceptibility in Han Chinese warrant the use of CCN4 as a diagnostic marker to stratify individuals at risk of developing RA, and CCN4 might serve as a potential target in RA disease [12]. Meanwhile, another study demonstrates that miR-515-5p could inhibit *WISP1* gene expression in human RA FLS [75], but the function of CCN4 in RA is not well understood yet.

In summary, CCN4 is harmful for OA, and *Ccn4* genetic polymorphisms are associated with RA susceptibility. A genetic variation in the *WISP1* gene locus is associated with spinal OA [72] and certain genes have been implicated in OA pathophysiology [73]. CCN4 stimulation of human OASFs increases VCAM-1 expression [39] and increases IL-6 production [74], while increases in Wnt signaling and WISP1 expression are linked to OA pathology [17,19,20]. In RA, *WISP-1* polymorphisms have been linked to RA susceptibility in Han Chinese [12], while miR-515-5p inhibits *WISP-1* gene expression in human RA FLS [75].

## 6. The Role of CCN5 in RA and OA

In normal human hip synovium samples, CCN5 is moderately expressed in the superficial layers, matrix and perivascular cells, whereas in samples taken from patients with advanced RA or OA, CCN5 expression is strong in RA and OA knee and hip synovium, but minimal in RA and OA cartilage [47]. Interestingly, real-time quantitative-polymerase chain reaction (qPCR) analysis of *WISP2* expression in arthritic synovial tissues by Tanaka and colleagues detected preferential expression of *WISP2* mRNA in all five human RA synovial tissue samples, compared with just one of four human OA synovial tissues [22], whereas a later qPCR analysis found *WISP2* mRNA and protein expression in human OA synovial synovium, infrapatellar fat pad tissues and human primary chondrocytes, as well as significantly higher *WISP2* mRNA expression in OA infrapatellar fat pads compared with samples from healthy controls [76]. Tanaka and colleagues also observed dose- and time-dependent upregulation of *WISP2* by estrogen in RASFs, which was substantially increased when RASFs were activated by Wnt signaling in the presence of estrogen [22]. In an examination of bone phenotype of adult CCN5/WISP5 knockout (*Ccn5^LacZ/LacZ^*) mice, loss of *Ccn5* did not appear to affect trabecular bone mineral density (BMD), bone volume fraction (BV/TV) or cortical bone thickness [24]. However, the study researchers noted that while these mice do not exhibit a discernable phenotype, it does not necessarily mean that *Ccn5* is unimportant in bone homeostasis; for instance, *Ccn3/Nov* knockout mice do not show an overt skeletal phenotype, but their bone healing is accelerated after injury [24]. In view of the limited reports, more research is needed to fully characterize how *Ccn5* affects synovitis in OA and RA.

In summary, CCN5 expression is increased in OA and RA disease. *WISP2* mRNA expression is significantly increased in human OA infrapatellar fat pad samples compared with healthy tissue samples [76]. Interestingly, an investigation into the biological role of CCN5 has reported that CCN5 is not required for normal bone formation, although it is necessarily unimportant in bone biology, since *Ccn3/Nov* knockout mice lack an overt skeletal phenotype but exhibit accelerated bone healing after injury; this might also be the case for *Ccn5* [24]. In RA, *WISP2* is synergistically upregulated in RASFs by estrogen and WNT pathways, which suggests that WISP2 is involved in the pathology of the disease [22].

## 7. The Role of CCN6 in RA and OA

Investigations into the mode of action of WISP3 and its function during cartilage growth and maintenance have ascertained that WISP3 is an important structural component of cartilage [41]. WISP3 is secreted from chondrocyte lines, while pure recombinant WISP3 protein appears to function as a ligand and signals via autocrine and/or paracrine modes [41]. Besides regulating collagen II and aggrecan expression, WISP3 may also contribute to cartilage growth and maintenance by promoting superoxide dismutase (SOD) expression and activity in chondrocytes [41]; such activity is very important for sustaining tissue homeostasis under conditions of cellular hypertrophy that may contribute to cartilage degeneration and the development of degenerative joint disease [77]. Interestingly, Lamb and colleagues have speculated that the functional consequences of WISP3 secretion could be impaired by disruption of the signal peptide that flanks the associated WISP3*84AA SNP within intron 1 [23]. More clarification is needed on this aspect.

CCN6 expression is generally undetectable in hip and knee joint tissues from patients with advanced RA or OA, and is minimal in the multilayered synovial cells from RA and OA knees [47], although evidence of high WISP3/CCN6 expression in end-stage OA cartilage suggests that CCN6 has a role in cartilage homeostasis [42]. It is speculated that the high expression of WISP3/CCN6 in end-stage OA cartilage may reflect attempts by cartilage to inhibit aggrecan breakdown and prevent further cartilage damage [42]. Moreover, the overexpression of WISP3/CCN6 in immortalized chondrocyte C-28/I2 cells is associated with substantially reduced levels of ADAMTS-4 and ADAMTS-5 expression, whereas MMP-1 and MMP-10 expression is increased, while gene silencing of WISP3/CCN6 in cytokine-stimulated primary chondrocytes enhances ADAMTS-5 expression and suppresses MMP-10 expression, suggesting that CCN6 has anticatabolic effects [42].

Higher levels of *WISP3* mRNA have been observed in RA synovium and FLS compared with OA and normal synovial tissue, and proinflammatory cytokines can further increase *WISP3* mRNA expression in RA FLS [21]. Intriguingly, similar levels of WISP3 protein expression in RA, OA and normal synovium suggest a lack of coordinated regulation between WISP3 protein and mRNA [21]. Interestingly, WISP3 appears to have an important role in the development of juvenile idiopathic arthritis (JIA), a group of chronic inflammatory arthropathies of childhood with onset before the age of 16 years [78]. Much research has explored the genetic basis of JIA, but the etiology of this disease is still not well understood [79,80]. Early initiation of disease-modifying antirheumatic drugs (DMARDs) is advised by the American College of Rheumatology (ACR) for pediatric patients with JIA [81]. One analysis of *WISP3* SNPs in blood samples obtained from two independent cohorts of patients with polyarticular-course JIA (≥5 joints involved) that included diagnoses of extended oligoarthritis, RF-negative polyarthritis and RF-positive polyarthritis, found replication of a positive association with an SNP within the first intron of the *WISP3* gene (WISP3*G84A) [23]. Individuals homozygous (AA) for G84A had a two-fold higher risk for polyarticular-course JIA compared with those who were not AA homozygous [23].

In summary, CCN6 is possibly beneficial for OA. High WISP3/CCN6 expression in end-stage OA cartilage suggests that CCN6 contributes to cartilage homeostasis [42]. Moreover, CCN6 is involved in complex context-dependent roles in cartilage biology, according to the evidence showing that WISP3/CCN6 mediates metalloproteinase expression through different pathways and modulates various signaling cascades [42]. Not only is *WISP3* gene expression in RA synovium and FLS markedly higher than that in OA and normal synovial tissue, but WISP3 mRNA expression is significantly increased in RA FLS when stimulated by proinflammatory cytokines [21]. Finally, replication of a positive association with a polymorphism within the first intron of the *WISP3* gene increases the risk of developing polyarticular-course JIA [23].

## 8. Conclusions

Several lines of evidence suggest that it is worthwhile to target the different members of the CCN family in both OA and RA disease. However, because of different effects of CCN proteins in RA and OA, an individualized approach with these CCN proteins for the management of these arthritis disorders should be considered in future applications.

## Figures and Tables

**Table 1:** Evidence regarding the involvement of CCN proteins in RA and OA.

CCN Protein	Disease	Targets	Target Factors		Results	References
**CCN1**	OA	osteoblast	IL-6, oncostatin M	↑	inflammation	[26]
	OA	cartilage	ADAMTS-4	↓	chondrocyte cloning	[15,27,28]
	RA	synovial fluid	CCL2	↑	inflammation	[11]
	RA	RA FLS	IL-1β, IL-6, IL-17	↑	inflammation	[29,30,31]
	RA	osteoblast	VEGF	↑	angiogenesis	[32]
	RA	RA FLS	MMP-3	↑	inflammation	[33]
**CCN2**	OA	OASF	CCL2/MCP-1	↑	inflammation	[34]
	OA	cartilage	Wnt-β-catenin	↑	chondroprotective	[35]
	RA	RASF	M-CSF, RANKL	↑	osteoclastogenesis	[36]
	RA	RA FLS	VEGF	↑	angiogenesis	[37]
**CCN3**	OA	cartilage	IL-1β	↓	protective effect	[38]
**Other CCNs**						
**CCN4**	OA	OASF	VCAM-1	↑	monocyte adhesion	[39]
	OA	synovium, cartilage	MMPs	↑	synovium & cartilage damage	[40]
**CCN6**	normal	chondrocyte	collagen II, aggrecan, SOD	↑	cartilage growth	[41]
	OA	chondrocyte	ADAMTS-4, ADAMTS-5	↓	anticatabolic effects	[42]
			MMP-1, MMP-10	↑		

RA, rheumatoid arthritis; OA, osteoarthritis; IL-6, interleukin 6; ADAMTS-4, a disintegrin and metalloproteinase with thrombospondin motif 4; CCL2, chemokine ligand 2; FLS, fibroblast-like synoviocyte; VEGF, vascular endothelial growth factor; MMP, matrix metallopeptidase; OASF, osteoarthritis synovial fibroblast; MCP-1, monocyte chemoattractant protein-1; RASF, rheumatoid arthritis synovial fibroblast; M-CSF, macrophage colony-stimulating factor; RANKL, receptor activator of nuclear factor-kappa B ligand; VCAM-1, vascular cell adhesion molecule-1; SOD, superoxide dismutase.

## Data Availability

Not applicable.
